# Navigating the Landscape of CMT1B: Understanding Genetic Pathways, Disease Models, and Potential Therapeutic Approaches

**DOI:** 10.3390/ijms25179227

**Published:** 2024-08-26

**Authors:** Mary Kate McCulloch, Fatemeh Mehryab, Afrooz Rashnonejad

**Affiliations:** 1 Center for Gene Therapy, The Abigail Wexner Research Institute at Nationwide Children’s Hospital, 575 Children’s Crossroad, Columbus, OH 43215, USA; 2 Molecular, Cellular, and Developmental Biology Program, The Ohio State University, Columbus, OH 43210, USA

**Keywords:** Charcot–Marie–Tooth type 1B, neuropathy, schwann cells, myelin protein zero, gene therapy

## Abstract

Charcot–Marie–Tooth type 1B (CMT1B) is a peripheral neuropathy caused by mutations in the gene encoding myelin protein zero (MPZ), a key component of the myelin sheath in Schwann cells. Mutations in the *MPZ* gene can lead to protein misfolding, unfolded protein response (UPR), endoplasmic reticulum (ER) stress, or protein mistrafficking. Despite significant progress in understanding the disease mechanisms, there is currently no effective treatment for CMT1B, with therapeutic strategies primarily focused on supportive care. Gene therapy represents a promising therapeutic approach for treating CMT1B. To develop a treatment and better design preclinical studies, an in-depth understanding of the pathophysiological mechanisms and animal models is essential. In this review, we present a comprehensive overview of the disease mechanisms, preclinical models, and recent advancements in therapeutic research for CMT1B, while also addressing the existing challenges in the field. This review aims to deepen the understanding of CMT1B and to encourage further research towards the development of effective treatments for CMT1B patients.

## 1. Introduction

Charcot–Marie–Tooth type 1B (CMT1B) is a demyelinating neuropathy caused by mutations in the myelin protein zero (*MPZ*) gene [1,2,3]. CMT1B phenotypes are variable in severity and can manifest early in infancy or childhood with symptoms including muscle weakness, foot deformities, sensory loss, and pain. This disease can also develop late into adulthood with symptoms including muscle atrophy, weakness, and nerve pain [4,5,6,7]. Currently, there are no therapeutics available to treat this peripheral neuropathy; however, several promising approaches are under investigation and will be discussed in this review.

Mutations in the *MPZ* gene are the main culprit of CMT1B. MPZ proteins adhere to each other through homophilic interactions on Schwann cell membranes, facilitating the stacking of the multiple layers of myelin lamellae [8,9]. Additionally, MPZ aids in myelin compaction by interacting with other myelin proteins such as myelin basic protein (MBP) and peripheral myelin protein 22 (PMP22) [8,10]. Furthermore, it participates in signal transduction pathways that regulate myelin formation and maintenance, ensuring the structural integrity and functionality of the myelin sheath [8]. Some of the most common mutations in *MPZ*, such as Arg98Cys and Ser63 deletion, are employed in disease models to replicate the CMT1B phenotype [11,12,13,14,15,16,17,18,19,20].

Gene therapy represents a promising therapeutic approach for various forms of CMT, with successful preclinical studies already conducted for several subtypes [4,21,22,23,24,25,26,27,28,29,30,31,32,33,34,35]. However, gene therapy studies for CMT1B have yet to emerge. To develop effective treatments for this neuropathy, it is essential to understand the genetic mechanisms underlying the disease, create accurate disease models for therapeutic testing, and establish robust outcome measures to assess therapeutic efficacy within study protocols. Herein, an overview of recent developments in the field is provided, and challenges and future directions in CMT1B research toward an efficacious treatment are further described.

## 2. Genetic and Mechanism

CMT1B is caused by over 200 mutations in the gene encoding MPZ, also known as P0 [1,2,3]. The MPZ protein is a 27-kDa single membrane glycoprotein that constitutes the primary protein of the peripheral nerve myelin sheath, comprising 50% to 60% of its total protein content. This glycoprotein plays a pivotal role in compacting the layers of myelin [8,9,36,37,38,39]. It is primarily expressed in myelinating Schwann cells, and at much lower basal levels during the onset of embryonic Schwann cell development from the neural crest [40,41,42,43]. The MPZ protein consists of an extracellular domain, a transmembrane domain, and a short cytoplasmic tail. The extracellular domain has adhesive properties crucial for the formation and stabilization of the compact myelin structure [39,44].

Although the genetic mechanism of CMT1B is not fully understood, it is known that most mutations in the *MPZ* gene cause neuropathy through a toxic gain of function by the mutant protein [7]. This can occur through different mechanisms such as endoplasmic reticulum (ER) retention, activation of the Unfolded Protein Response (UPR), mis-glycosylation and disruption of myelin compaction, and mistrafficking of mutant MPZ to the myelin sheath [7,8,45,46]. Additionally, a few mutations are also associated with a loss of function pathway in this disease, leading to milder neuropathy phenotypes [3].

The ER plays a crucial role in protein synthesis, folding, modification, and transport within cells, and disruptions in these processes can contribute to disease. Upon translation, proteins will be folded in the ER lumen, which is facilitated by chaperone proteins [47]. Post-translational modifications can also take place in the ER, which is necessary for proper protein transport. The ER is also a major center for quality control upon protein synthesis. If misfolded proteins accumulate in the lumen, ER stress is induced, and molecular chaperones can detect misfolded proteins to activate signaling pathways to combat this stress [47,48]. In healthy cells, MPZ is processed in the ER of Schwann cells and then sorted for vesicle transport by the Golgi apparatus [49]. However, some mutations in *MPZ* cause misfolding of mutant proteins, leading to their retention in the ER rather than transport to the myelin sheath of Schwann cells. This accumulation of mutant MPZ in the ER lumen triggers ER stress and can disrupt the signaling of pathways such as the neuregulin 1 (Nrg1) /ErbB pathway [50], and can also activate pathways such as ER-associated degradation (ERAD) [51] and the UPR [7]. For example, studies on mice demonstrated that *Mpz* R98C mutation causes the protein to be retained in the endoplasmic reticulum (ER) of Schwann cells, triggering an unfolded protein response. This response leads to developmental arrest of Schwann cells at the promyelinating stage and delays their development. Mutant Schwann cells show increased expression of c-Jun, an inhibitor of myelination, and decreased expression of the promyelinating transcription factor Krox-20. These disruptions result in impaired Schwann cell development and demyelination, ultimately leading to the clinical manifestations of CMT1B disease [12].

**Figure 1:**
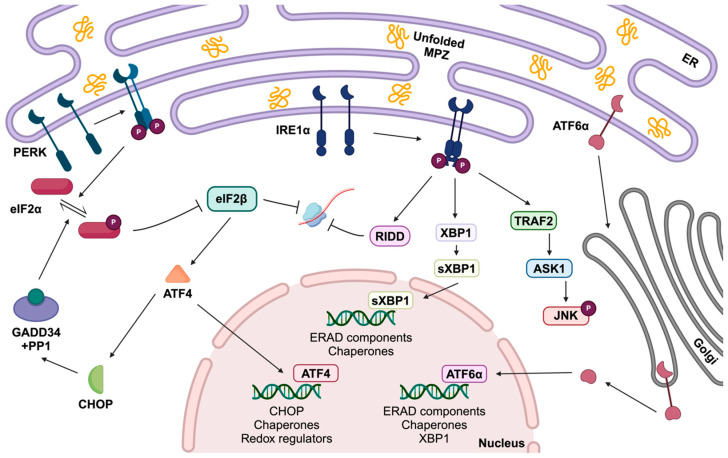
Schematic illustration of the UPR in CMT1B. Unfolded mutant proteins accumulate in the ER and are sensed by the ER stress sensors PERK, IRE1α, and ATF6α, leading to the activation of the UPR. Upon detecting misfolded proteins, PERK dimerizes and phosphorylates eIF2α, resulting in the upregulation of ATF4, CHOP, and GADD34. ATF4 stimulates CHOP production, a transcription factor that promotes apoptosis under unresolved ER stress conditions. If the UPR successfully resolves ER stress, GADD34 forms a complex with PP1, acting as a negative feedback mechanism to restore normal translation. IRE1α, another stress transducer, autophosphorylates upon sensing misfolded proteins and exhibits endoribonuclease activity, splicing XBP1 mRNA transcripts. The spliced XBP1 (XBP1s) produces a transcription factor that translocates to the nucleus, inducing ERAD. Finally, ATF6α translocates to the Golgi apparatus, where it is cleaved by proteases. The cleaved ATF6α is then transported to the nucleus to upregulate the production of ERAD components, chaperone proteins, and XBP1 transcripts [48,52]. Abbreviations: ATF4: activating transcription factor 4; ATF6α: activating transcription factor 6 alpha; CHOP: C/EBP homologous protein; eIF2α: eukaryotic initiation factor 2 alpha; eIF2β: eukaryotic initiation factor 2 beta; ER: endoplasmic reticulum; ERAD: endoplasmic reticulum associated degradation; GADD34: growth arrest and DNA damage-inducible protein 34; IRE1α: inositol-requiring enzyme 1 alpha; MPZ: myelin protein zero; PERK: protein kinase RNA-like endoplasmic reticulum kinase; PP1: protein phosphatase 1; XBP1: X-box binding protein 1.

The UPR is a canonical signaling pathway activated by stress transducers upon detection of ER stress. UPR components function as a critical axis integrating both stress response and metabolic pathways. Protein kinase RNA-like ER kinase (PERK), activating transcription factor 6 (ATF6), and inositol-requiring enzyme 1 (IRE1) are all transmembrane proteins that can be activated during the UPR and are capable of sensing accumulated proteins in the ER. Signals from stress transducers can lead to decreased protein synthesis, increased protein degradation, expansion of the ER membrane, and regulation of protein influx into the ER to prevent overload [47,48,52] (Figure 1).

Protein chaperone binding immunoglobulin protein (BIP) is crucial in stress detection, and consequently, activation of stress transducers. In normal conditions, BIP is bound to the luminal domains of PERK and IRE1 alpha (IRE1α) to inhibit dimerization. However, BIP preferentially binds to misfolded proteins, so under stress conditions, BIP will dissociate from these transducers, which allows for autophosphorylation, dimerization, and finally activation of these signaling pathways [48]. Following PERK autophosphorylation, PERK phosphorylates eukaryotic initiation factor 2 alpha (eIF2α) at the S51 position of the alpha subunit to activate downstream signaling events [47,48]. eIF2α then complexes with eIF2 beta (eIF2β) to inhibit function, which leads to the translation of ATF4. ATF4 stimulates the production of C/EBP homologous protein (CHOP) and in turn induces growth arrest and DNA damage-inducible protein 34 (GADD34) expression. GADD34 is a regulatory phosphatase subunit complexing with protein phosphatase 1 (PP1), which is responsible for the de-phosphorylation of eIF2α and therefore is important in negative feedback regulation. CHOP also serves as a transcription factor for the expression of apoptotic genes [47,48,51].

IRE1α possesses both kinase and endoribonuclease activities. Upon BIP dissociation, IRE1α dimerizes and autophosphorylates, leading to activation of the molecule’s RNase domain. IRE1α processes *X-box binding protein 1* (*XBP1*) mRNA transcripts and excises a 26-nucleotide-long intron, resulting in a spliced and truncated version of the transcript (*XBP1s*) [48]. *XBP1s* encodes a transcription factor that is shuttled to the nucleus, where it induces the expression of genes involved in ERAD [48].

ATF6 is also bound by BIP, which blocks its localization signal to the Golgi apparatus. Upon BIP dissociation, ATF6 interacts with vesicle-generating proteins and is translocated to the Golgi [48]. In the Golgi, ATF6 is cleaved by proteases in its transmembrane domain, resulting in a cleaved cytosolic fragment of ATF6 being translocated to the nucleus. Once in the nucleus, cleaved ATF6 is able to upregulate the expression of ERAD components, chaperone proteins, and XBP1 [47,48].

As described above, three main indicators are employed to identify the activation of each arm of the UPR: (1) PERK: Upregulation of transcription factor CHOP and translocation to the nucleus, (2) ATF6: Cleavage of ATF6, and (3) IRE1: Splicing of *XBP1* mRNA. Mutant MPZ has been shown to activate all arms of the UPR [12,14,52,53]. However, misfolding of proteins or activation of the UPR does not occur in all *MPZ* mutations, which indicates that this is not the sole genetic mechanism underlying CMT1B. Mis-glycosylation, involving either the addition of a new glycosylation site or the removal of an essential site, has also been proposed as a pathomechanism for CMT1B. This disruption in glycosylation has been shown to impair protein transport, as well as the adhesive properties of MPZ by interfering with immunoglobulin-like (Ig-like) domain positioning and consequently amino acid interactions [45,46].

In short, PERK phosphorylates eIF2α to diminish protein synthesis in ER stress conditions. It can result in selective mRNA translation for ATF4, responsible for the activation of CHOP and GADD34 [54] (Figure 1). D’Antonio et al. [19] showed that the PERK-CHOP arm is pathogenic in the CMT1B mouse model, and the pharmacological or genetic limitation of GADD34 could augment phosphorylated eIF2α, reduce misfolded protein accumulation in ER, and thus improve myelination [19]. The same research team continued to study the pathogenic mediators of CMT1B and conversly suggested that PERK ablation mitigated CMT1B neuropathy. Their data indicated the involvement of other mechanisms in the disease symptoms [17], and also revealed that the harmful effect of PERK is Schwann cell-specific [18]. Furthermore, another study demonstrated that eIF2α phosphorylation is vital; however, the absence of phosphorylation did not significantly affect the ER stress level. Accordingly, it was proposed that the UPR mechanism could be different in Schwann cells at some points [54]. Moreover, in a recent study published in bioRxiv, the role of IRE1α/XBP1 was explored, and ameliorated myelination was observed via the activation this pathway in CMT1B dorsal root ganglion (DRG) explants [16].

**Figure 2:**
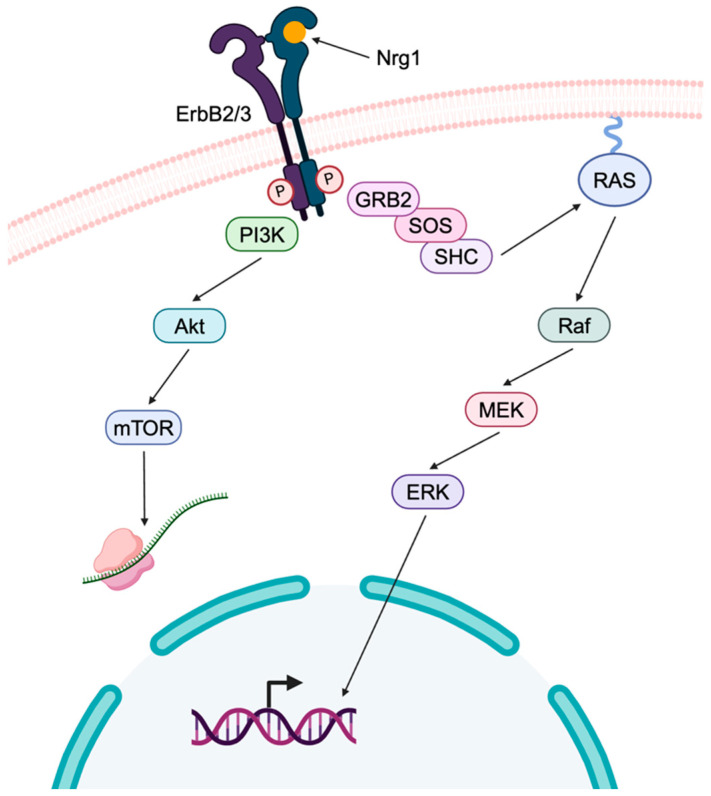
Schematic illustration of Nrg1/ErbB signaling. Nrg1 binds to ErbB2/3 heterodimer receptors on the cell surface, leading to the activation of the PI3K/Akt signaling pathway. This activation stimulates mTOR, which regulates protein translation. Concurrently, GRB2, SOS, and SHC bind to phosphorylated tyrosine residues, activating the GTPase RAS and the kinase Raf. Raf subsequently initiates the MEK/ERK signaling pathway, culminating in the regulation of gene expression in the nucleus [50,55,59]. Abbreviations: Akt: protein kinase B; ErbB: Erb-B2 receptor tyrosine kinase 2; ERK: extracellular signal-regulated kinase; GRB2: growth factor receptor-bound protein 2; GTPase: guanosine triphosphatases; MEK: mitogen-activated protein kinase; mTOR: mammalian target of rapamycin; Nrg1: neuregulin 1; PI3K: phosphoinositide 3-kinase; Raf: rapidly accelerated fibrosarcoma; RAS: Rat sarcoma; SOS: Son of sevenless.

Additionally, Nrg1/ErbB signaling (Figure 2) has been shown to play a major role in regulating the myelination of axons by Schwann cells, and disruptions have been implicated in CMT pathogenesis. Within the ErbB family, Schwann cells only express ErbB2 and ErbB3 tyrosine kinase receptors, both of which are necessary as ErbB2 does not possess the ability to bind ligands such as Nrg1 and ErbB3 lacks kinase activity [50]. In Schwann cells, Nrg1 type III binds to the ErbB2/3 heterodimer, which activates pathways to promote myelination. Upon ligand binding of ErbB2/3, PI3K is activated, which in turn activates Akt, a kinase that promotes Schwann cell growth and survival. Akt is then capable of inhibiting a negative regulator of the mTOR pathway. Following activation, mTOR promotes translation of various proteins, including MPZ [55,56,57,58,59].

Nrg1/ErbB signaling can also stimulate mitogen-activated protein kinase (MEK)/ extracellular signal-regulated kinase (ERK) pathway activation. Activation of this pathway occurs when GRB2 binds to phosphorylated ErbB receptors inside the Schwann cell and recruits Son of sevenless (SOS) and SHC molecules, which activate guanosine triphosphatases (GTPase) Rat sarcoma (RAS) and rapidly accelerated fibrosarcoma (Raf) kinase. Phosphorylation of MEK and ERK via Raf results in the translocation of ERK to the nucleus where it modulates the expression of myelination genes [50,55]. The connection between defects in these signaling pathways and the development of CMT1B is not well understood, but has been investigated in other demyelinating types of CMT [49,50,56,60]. In a recent study, Nrg1 type III was genetically overexpressed to further activate PI3K/Akt and MAPK/Erk pathways, and it was demonstrated that this overexpression could ameliorate CMT1B neuropathy in vivo [56]. It is believed that the Neuregulin pathway modulates myelin thickness and favors myelination by the activation of those critical downstream signaling pathways in Schwann cells [61] (Figure 2).

**Figure 3:**
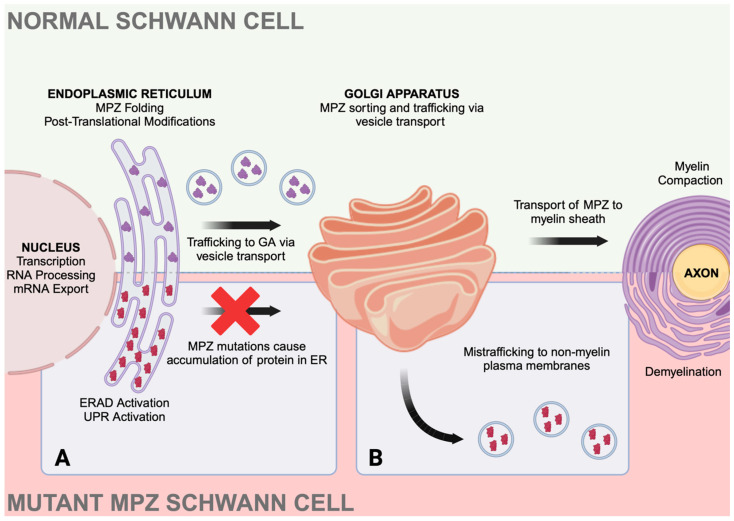
Summary of biogenesis and trafficking of MPZ in healthy Schwann cells compared to pathomechanisms in CMT1B Schwann cells. The upper half of the figure illustrates the normal biogenesis and trafficking of MPZ in healthy Schwann cells. *MPZ* mRNA is processed in the nucleus and exported to the cytoplasm, where it is translated. Properly folded and modified MPZ proteins (depicted in purple) enter the ER, are transported in vesicles to the Golgi apparatus for further processing and sorting, and are ultimately trafficked to the plasma membrane of myelinating Schwann cells [8]. The lower half of the figure depicts two potential pathways by which *MPZ* mutations can lead to demyelination, resulting in CMT1B. (**A**) shows that mutations in *MPZ* can cause protein misfolding, leading to the accumulation of mutant MPZ proteins (depicted in red) in the ER. This accumulation can trigger ER stress and activate the UPR and ERAD pathways [7,51]. (**B**) illustrates an alternative scenario where mutant MPZ proteins are correctly transported to the Golgi but are then misrouted and trafficked to the plasma membranes of cell types other than myelinating Schwann cells [8,63]. Both pathogenic mechanisms have been observed to contribute to the development of the CMT1B phenotype. Abbreviations: ER: endoplasmic reticulum; ERAD: endoplasmic reticulum-associated degradation; GA: Golgi apparatus; MPZ: myelin protein zero; mRNA: messenger RNA; UPR: unfolded protein response.

Another possible mechanism underlying CMT1B could involve the mistrafficking of mutant MPZ to the myelin sheath [8,62] (Figure 3). In a study developing a P0Q215X knock-in mouse model, Fratta et al. [63] showed that Mpz with the Q215X mutation was not being retained in the ER, and instead was found to be trafficked primarily to the myelin sheath while also being detected in non-myelin plasma membranes surrounding Schwann cells. Impaired membrane targeting of MPZ to non-myelin membranes could play a role in defective axonal sorting, contributing to the disease phenotype [63].

## 3. Disease Models

The ability to model a disease is crucial for investigating the underlying mechanisms of disease pathology, exploring potential therapeutic targets, and testing the efficacy of novel treatments. Given the complex nature of CMT1B, utilizing a variety of disease models is imperative.

While in vitro models offer a controlled system for investigating the cellular and molecular mechanisms underlying CMT1B pathogenesis, animal models provide valuable insights into the physiological and behavioral aspects of the disease in complex organisms. This allows researchers to study disease progression and test therapeutic interventions in vivo. By employing a diverse array of disease models, researchers can gain a comprehensive understanding of CMT1B and identify promising therapeutic strategies to improve the lives of individuals affected by this debilitating disorder.

### 3.1. In Vitro Models

There are several in vitro models used to replicate the expression of mutant MPZ present in CMT1B. In vitro models of CMT1B have allowed researchers to identify new pathomechanisms, elucidate signaling pathways involved in the disease, and screen for potential therapeutics for treatment. In vitro models offer the ability to tightly control and monitor experimental conditions, while also being cost-effective. In vitro models are widely used to test early hypotheses and conduct screenings before moving to the use of in vivo models.

Although immortalized cell lines stably or transiently expressing various MPZ mutations [14,45,62,64] have been utilized to study CMT1B, researchers are increasingly adopting DRG explant or DRG/Schwann cell co-cultures [15,16,17,45,56] as a more physiologically relevant system for investigating peripheral neuropathy. DRG cultures contain primary cells that can maintain the genotype of the organism they were isolated from and allow for visualization of myelination processes in certain conditions [65].

The CMT field is also benefiting from patient-derived induced pluripotent stem cells (iPSCs), which offer the advantage of patient specificity and can differentiate into various cell types, including Schwann cells, or form organoids as more complex in vitro models. Although two iPSC lines have been established for CMT1B mutations [66,67], they have yet to be widely created for other mutations or utilized as valuable models in future research. An overview of the studied in vitro disease models is presented in Table 1.

#### 3.1.1. Immortalized Cell Lines

Both HeLa and HEK293 cell lines transiently expressing mutant MPZ were used in a study investigating the therapeutic effects of curcumin [64]. In another study, HeLa cells with mutations indicative of both early and late-onset phenotypes were utilized to investigate the pathomechanisms of MPZ mutations [62]. Furthermore, RT4 Schwann cells isolated from the sciatic nerve tumors of BDIX strain rats [69,70] maintain the expression of key genes characteristic of Schwann cells, making them an ideal platform for studying Schwann cell biology [69,71,72]. Due to these characteristics, researchers used RT4-D6P2T cells to create stable cell lines expressing S63del mutant *MPZ* associated with CMT1B phenotypes [68]. This model facilitates the screening of potential therapeutic compounds for restoring myelin integrity and Schwann cell function in CMT1B [68]. Although RT4-D6P2T offers advantages similar to other immortalized cell lines, it is more relevant for peripheral nerve research. However, it has a significant limitation: the absence of Nrg1/ErbB system expression, which is present in native Schwann cells. This system is crucial for the development of Schwann cell precursors and for specific interactions between axons and Schwann cells [73].

#### 3.1.2. Induced Pluripotent Stem Cells

iPSCs have emerged as a powerful tool in CMT1B research. iPSCs were first established by Takahashi and Yamanaka [74], in which they reprogrammed differentiated cells from mouse embryos and adult fibroblasts by introducing *Oct3/4*, *Sox2*, *c-Myc*, and *Klf4* [74]. In 2017, Son et al. [66] produced an iPSC line from an 81-year-old patient with an *MPZ* S140T (c.T>A) mutation. Lymphoblastoid cell lines (LCLs) were collected from the patient and reprogrammed through electroporation of plasmids containing *Oct4*, *Sox2*, *Klf4*, *l-Myc*, *Lin28m* and *p53*-targeting shRNA [66]. In 2019, Xu et al. [67] also developed a human iPSC cell line from samples taken from a CMT1B patient. Renal epithelial cells were obtained from a 2-year-old patient with a c.292C>T point mutation in *MPZ*, resulting in the substitution of arginine at position 98 with cysteine (R98C). Patient urinary cells were reprogrammed through the use of retroviral vectors containing *Oct4*, *Sox2*, *KLF4*, and *c-Myc* [67]. Although this model has not been commonly used for CMT1B, Shi et al. [75] developed human iPSC lines from CMT1A patients as in vitro models by inducing differentiation into Schwann cells via the neural crest stem cell stage. However, the authors noted that the differentiation was severely impaired, and the cells tended to differentiate into endoneurial fibroblast-like cells [75]. Another emerging model is an iPSC-derived organoid culture containing different cell types, mimicking the peripheral nerve environment [76]. This has great potential, as it would best mimic the complexity of tissues in vivo. The success of these models in CMT1A research indicates that they would be useful in modeling CMT1B. Advantageously, iPSC-derived neural cells isolated from patients maintain the genotype and are more indicative of the phenotype of the disease. However, a significant hurdle in utilizing iPSCs is the lack of standardized protocols for efficiently differentiating iPSCs into fully functional myelinating Schwann cells. While some protocols exist for deriving Schwann cells from iPSC-derived neural crest stem cells, they often result in impure cultures and require several months to generate functional Schwann cells [77]. Taken together, these studies suggest that additional efforts are required to optimize the differentiation of iPSCs into Schwann cells and organoids, as they have the potential to serve as a valuable model for studying CMT1B.

#### 3.1.3. DRG Explants and DRG/Schwann Cell Co-Cultures

DRGs are clusters of primary neuron cell bodies located bilaterally to the spine and are responsible for transmitting sensory information from the peripheral nervous system to the central nervous system [78]. DRGs are composed of several different cell types, including sensory neurons, neuronal progenitors, satellite glial cells, Schwann cells, and stem cells [65,79]. Isolating and culturing DRGs provides a valuable disease model for CMT1B, a primary cell model mimicking the disease phenotype in vitro with the ability to examine myelinated axons in culture.

**Figure 4:**
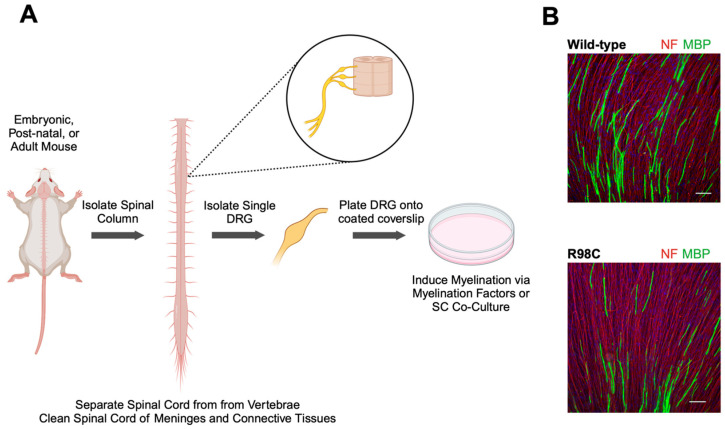
Overview of myelinating DRG culture preparation. (**A**) Embryonic, post-natal, or adult mice are dissected to isolate the spinal column. Vertebrae are removed from the spinal cord, followed by the removal of extra tissues, including meninges and connective tissues, to obtain a clean spinal cord with bilateral DRGs. Singular DRGs are isolated by pulling the tissue just under the DRG closest to the spinal cord and then placed onto pre-coated coverslips to promote adhesion. Efficient myelinating DRG cultures require the addition of myelination factors or co-culture with Schwann cells isolated and purified from sciatic nerves. (**B**) Immunofluorescence staining of myelinating DRG cultures from wild-type and R98C mutant mice. DRG explants were incubated with anti-NF to label neurons and anti-MBP as a myelination marker. Explants from heterozygous R98C mice (bottom) show lower levels of myelination compared to those from wild-type mice, indicating successful recapitulation of the disease phenotype [84]. Scale bars represent 100 μm. Abbreviations: DRG: dorsal root ganglion; MBP: myelin basic protein; NF: neurofilament; SC: Schwann cell.

This culture method, illustrated in Figure 4, involves isolating DRGs from embryonic, postnatal, or adult animals, plating onto a suitable culture substrate, and then inducing myelination with myelination-inducing factors such as ascorbic acid [56,80]. Following the plating of the explants, Schwann cells within the DRG will radiate outwards and proliferate on extending axons. Myelination is subsequently induced through the addition of specific factors, which is necessary due to the absence of endogenous myelination signals in cultured Schwann cells [81]. This method poses minimal risk of fibroblast contamination, and myelinating DRGs are obtained in a relatively short period of time [82].

Another option for a myelinating culture is isolating the primary neurons from DRGs, co-culturing with primary Schwann cells that have been isolated from the sciatic nerve, and again adding myelination-inducing factors [65,83]. In this culture system, also known as DRG/Schwann cell co-culture, neurons are purified and DRGs are then dissociated by incubation with trypsin and mechanical homogenization. This method is more technically challenging and time-consuming but could provide a more useful system for studying myelinating Schwann cells specifically. It is necessary to have an efficient recreation of the multi-cellular environment of the peripheral nervous system, particularly focusing on Schwann cells, which play a crucial role in myelination and maintenance of axonal integrity [83].

In CMT1B, mutations in the *MPZ* result in demyelination and subsequent axonal degeneration. In 2005, rat and mouse DRGs were used in a study to identify the role of Nrg1 type III in myelination [57], proving the usefulness in this model in studying myelination and Schwann cells. More recently, DRG/Schwann cell co-cultures were used in a study on hyper-glycosylation of MPZ as a pathomechanism for CMT1B [45]. Furthermore, Bai et al. [15] used a myelinating DRG explant Schwann cell co-culture model to study the potential of IFB-088 treatment for CMT1B. Most recently, Touvier et al. [16] utilized a myelinating DRG explant model harvested from embryos at embryonic day 13.5 to examine the role that IREIα arm activation and XBP1 signaling play in myelination in CMT1B. They used DRG explants to test IRE1/XBP1 activating (IXA) compounds for therapeutic potential, as these compounds cannot pass the blood–brain barrier or the blood–nerve barrier.

By inducing myelination or co-culturing DRG neurons with Schwann cells isolated from CMT1B animal models, it is possible to study various aspects of CMT1B pathogenesis, including aberrant myelination, Schwann cell–axon interactions, and the effects of potential therapeutic interventions. Additionally, advancements in cell culture techniques and imaging modalities enable researchers to assess the efficacy of novel drugs or gene therapies aimed at ameliorating the symptoms of CMT1B.

### 3.2. In Vivo Models

Most in vivo models used for modeling CMT1B are knock-in mice with various mutations in the *Mpz* gene due to their genetic similarities with humans. Mice with mutations in the *Mpz* gene have been shown to have phenotypes similar to that of humans with CMT1B, such as progressive muscle weakness, muscle atrophy, motor impairment, and demyelination of peripheral nerves. Mouse models are typically characterized through morphological and electrophysiological analyses, as well as various behavioral assays. Histological staining of peripheral nerves can provide valuable knowledge about the pathology of the disease and the level of myelination. For example, the g-ratio is useful as it measures the degree of myelination by dividing the diameter of the axon by the outer diameter of the nerve fiber. Because CMT1B is a demyelinating neuropathy, models tend to have thinner myelin sheaths and therefore a higher g-ratio [12]. Motor nerve conduction velocity (MNCV) is an important electrophysiological measure that describes the speed of signal transmission through motor nerves. It is significantly reduced in CMT1B patients [85]. Compound muscle action potential (CMAP) is a measure of the action potentials signal elicited in muscle fibers immediately after nerve stimulation, which is also typically reduced in those affected by CMT1B [85]. Different *MPZ* mutations result in varying MNCV and CMAP values. We summarize several published models with their corresponding MNCV and CMAP data in Table 2.

Common behavioral assays for in vivo models involve testing motor function, muscle strength, balance, and overall activity. Relevant to CMT1B research, mice are tested via treadmill, rotarod, grid hang, and grip strength. CMT1B mice typically perform poorly compared to wild-type mice in these assays [6,12,45]. The most common in vivo mouse models in CMT1B research are further described in this section (Table 2).

#### 3.2.1. *Mpz* Null Mouse

*Mpz* knockout mice were generated by Giese et al. [86]. Null mice developed a tremor around two weeks of age, and progressively developed convulsions and dragging of their hindlimbs. Some mice also shrieked and mutilated their hindlimbs, demonstrating clear signs of pain and distress. Upon examination of femoral and sciatic nerves, myelin was de-compacted and much thinner in appearance. Homozygous mice exhibited a much more severe phenotype compared to heterozygous mice. *Mpz* knockout mice have been used to study pathomechanisms of CMT1B [87]; however, researchers have shifted towards using transgenic mice with point mutations in *Mpz*, as these models may more accurately represent CMT1B patients.

#### 3.2.2. *Mpz* R98C Mouse

Mice with the Arg98Cys (R98C) mutation in the *Mpz* gene are a common model used in modeling CMT1B. Saporta et al. [12] generated the knock-in mouse model by insertion of the mutation via homologous recombination in embryonic stem (ES) cells. This model shows phenotypes of varying severity, with symptoms including abnormal motor performance, significant reductions in motor nerve conduction velocity, and thinner myelination. The motor performance of both R98C homozygous and heterozygous mice was tested using rotarod at six weeks of age. Heterozygous mice performed poorly compared to wild-type mice with latencies of around 70 s as opposed to just over 100 s. Homozygous mice showed a much more severe phenotype and were unable to stay on the rotarod at all [12].

Electrophysiological analyses revealed that 6- to 8-week-old wild-type mice exhibited MNCVs of ~40 m/s. In contrast, both R98C heterozygous and homozygous mice showed significantly reduced MNCVs, measuring around 15 m/s and 4 m/s, respectively. CMAPs followed a similar trend, with wild-type mice demonstrating values of approximately 65 mV, R98C/+ mice at 38 mV, and R98C/R98C mice exhibiting the most severe reduction at 10 mV [12].

Morphological analyses of sciatic nerves from 6-week-old wild-type and mutant mice were conducted to assess the impact of the R98C mutation on the myelin sheath and evaluate the model’s relevance to CMT1B in humans. Wild-type mice exhibited an average g-ratio of 0.67, whereas R98C/+ mice demonstrated an increased average g-ratio of 0.77, indicative of a thinner myelin sheath. Due to the severity of demyelination in R98C/R98C mice, g-ratios could not be calculated for this group [12].

The authors performed X-ray diffraction analysis on sciatic nerve samples taken from wild-type, R98C/+, and R98C/R98C mice in order to determine differences in the amount of myelin present, myelin periods, and membrane packing abnormalities. Data showed that the scattering intensity from myelin was reduced in R98C/+ mice and even more drastically in R98C/R98C mice. Myelin periods were shown to slightly increase by <1% in heterozygous mice and by 9% in homozygous mice. Coherence lengths of myelin reduced by roughly 30% in heterozygous mice and had ~25% greater period distortions in contrast to wild-type mice. R98C/R98C mice had reduced coherence lengths by nearly 60% and period distortions double that of wild-type mice [12].

Observing an increased number of nuclei in the sciatic nerves of R98C mutant mice, Saporta et al. [12] employed bromodeoxyuridine (BrdU) analysis of nerves to quantify Schwann cell proliferation. BrdU-labeled nuclei were increased in both R98C/+ and R98C/R98C, but only the elevated levels in homozygous mice were statistically significant. Immunohistochemistry was also performed on the three genotypes at post-natal day 13 at the height of myelination to characterize Schwann cell development from promyelinating to myelinating cells in mutant mice. C-Jun, an inhibitory transcription factor, was significantly increased in both mutants, while activating transcription factor Krox20 levels only decreased significantly in homozygous mice [12].

Because mutant protein aggregation in the ER is heavily implicated as a pathomechanism in CMT1B, the authors investigated *Mpz* R98C’s effect by localizing Mpz expression in mutant nerves and found Mpz distributed in myelin but also surrounding the nucleus in R98C/+ mice. Only perinuclear Mpz was detected in homozygous mice [12]. Immunostaining of sciatic nerves from wild-type mice demonstrated that Mpz is localized to the myelin sheath, while the chaperone protein BIP is localized to the ER. In contrast, in homozygous mice both proteins were localized to the ER, indicating accumulation of mutant Mpz [12]. This accumulation of mutant Mpz in the ER led to a canonical UPR response, which was indicated by an increased level of cleaved ATF6 in both mutants, as well as an increase in CHOP levels determined by northern blot [12].

These mice have been used in several studies in recent years. Bai et al. [15] utilized *Mpz* R98C mice as a model to study the potential of IFB-088 as a therapeutic option through oral administration of either the vehicle or 1mg/kg of the compound twice a day starting at one month of age. Phosphorylated eIF2α levels were elevated in 1-month-old R98C/+ mice indicating a UPR response via PERK signaling. At three and five months of age, treatment with IFB-088 resulted in statistically significant improvements in grip strength, rotarod performance, and nerve conduction velocities. However, no significant differences in CMAP amplitudes were observed between wild-type and R98C/+ mice [15]. Morphological analysis revealed that after five months of IFB-088 treatment, quadriceps femoral nerves taken from R98C/+ mice showed reduced g-ratios and thicker myelin [15].

In another recent study, this model was utilized to investigate the involvement of the IRE1α/XBP1 signaling pathway in CMT1B. Touvier et al. [16] used R98C/+ mice as well as R98C mutant mice with *Xbp1* knocked out in Schwann cells (R98C/*Xbp1^SC-^*^KO^). Evaluation at six months of age showed that *Xbp1* ablation in Schwann cells severely worsened nerve conduction velocities and F-wave latencies compared to R98C/+ mice. R98C/*Xbp1^SC-^*^KO^ mice also performed more poorly on rotarod and had significantly increased g-ratios. These data indicate the protective role of this arm of the UPR [16].

#### 3.2.3. *Mpz* S63del Mouse

Deletion of Serine 63 is another mutation commonly used to mimic CMT1B in vivo. This transgenic mouse model was produced by Wrabetz et al. [7] via pronuclear injection into ES cells to study the pathomechanisms behind *Mpz* leading to CMT1B. The authors confirmed that this mutation acts through gain of function. To investigate mutant protein accumulation, the authors assessed BIP and CHOP mRNA and protein levels in sciatic nerves from S63del mice. They observed an increase in expression and the induction of sXBP1, indicating that mutant Mpz is retained in the ER and activates the UPR [7].

They observed impaired neuromuscular function similar to that of patients with CMT1B, which was confirmed by observing gait, posture, and tremor, and also by poor performances on the rotarod [7]. At four months of age, S63del mice were only able to stay on the rotarod roughly half as long as wild-type mice. Twelve-month-old mutant mice experienced demyelination and decreased MCNVs of ~24 m/s compared to ~43 m/s in wild-type mice of the same age, as well as atrophied muscle. Electrophysiological studies on sciatic nerve showed CMAP amplitudes in S63del mice of ~8.2 mV while wild-type mice were measured at ~14.1 mV. Furthermore, onion bulb formation and hypomyelination were observed in nerve sections, consistent with demyelinating neuropathy present in patients [7]. Musner et al. [17] reported that g-ratios of nerves taken from wild-type mice and S63del mice at four months of age were ~0.65 and ~0.69, respectively. From the same study, their data showed that ablation of PERK improved motor function in this model. Scapin et al. [51] used this model to study the role of eIF2α in the UPR and its relation to the development of CMT1B. They found that eIF2α plays a protective role in the Schwann cells of S63del mice, yet a lack of eIF2α did not majorly alter detected UPR levels. However, Schwann cell differentiation and myelination were dramatically delayed [51]. Pennuto et al. [53] utilized this transgenic model to further study the relationship between the transcription factor CHOP and neurodegeneration caused by the S63del mutation. They found that CHOP expression correlated with phenotype severity in these mice and that ablation of CHOP rescued motor function [53].

#### 3.2.4. *Mpz* D61N Mouse

The D61N mutation introduces a glycosylation site within the Ig-like domain, resulting in myelin malformation and partial retention of MPZ within the Golgi apparatus [8]. Veneri et al. [45] created a transgenic mouse model with this mutation using the Clustered Regularly Interspaced Short Palindromic Repeats (CRISPR)/Cas9 system to evaluate the effects of Mpz hyper-glycosylation in vivo. D61N mice developed a tremor around day 15 postnatal, and showed defective myelin compaction, with a g-ratio of ~0.72 compared to ~0.65 in wild-type mice. Three-month-old D61N mice exhibited impaired performance on the rotarod test, with an average latency period of ~184.7 s, in contrast to wild-type mice, which had an average latency period of around 329.7 s. Additionally, grip strength assessments conducted at three months of age revealed that wild-type mice generated an average force of ~97.19 g, while D61N mutant mice demonstrated a reduction in muscular strength, producing an average force of ~82.68 g [45].

Electrophysiological tests were performed at one and three months of age. At one month, D61N mice showed significantly reduced MNCVs of ~12.05 m/s compared to ~33.02 m/s in control mice. CMAPs were also severely reduced in mutant mice to roughly 0.2 mV whereas wild-type mice were measured at around 5.8 mV. At three months of age, the severity of the mutation’s effects was such that electrophysiological data could not be reliably recorded or were significantly impaired in the mice that underwent testing. This mutation resulted in a pronounced de/dysmyelinating phenotype in these transgenic mice [45].

While the molecular effects of the D61N mutation are still not well defined, the authors investigated the potential activation of the UPR in these mice. At one month of age, a slight but statistically significant increase in the expression of BIP and phosphorylated eIF2α in mutant sciatic nerves, compared to wild-type mice, suggests UPR activation. By six months of age, this increase was no longer detected. However, RNA-seq data revealed that pathways related to Schwann cell differentiation, proliferation, and extracellular matrix organization were disrupted, as indicated by the dysregulation of laminin, collagen, and cadherin families [45].

#### 3.2.5. *Mpz* I106L Mouse

Rünker et al. [89] generated an *Mpz* I106L transgenic mouse model via microinjection of zygotes to mimic a severe and early-onset form of CMT1B, and to investigate the pathomechanisms of the I106L mutation. An A→T nucleotide mutation was created in exon 3 of *Mpz*. Although the molecular mechanisms of this mutation have yet to be examined in depth, reverse transcription-polymerase chain reaction (RT-PCR) analysis revealed that transgenic *Mpz* is overexpressed by approximately six-fold compared to endogenous *Mpz* mRNA, which may contribute to the severe phenotype observed [89].

These mice presented with a tremor, muscle weakness, paw dragging, and inability to breed, likely due to impaired motor function. Adult I106L mice had MNCVs of around 2 m/s compared to 40.2 m/s in wild-type mice. CMAPs of plantar muscles in mutant mice (~1.1 mV) were also significantly lower than those of wild-type mice (~12.8 mV). They also had thinner myelin, onion bulbs, and tomacula in all nerve fibers [89]. Furthermore, at 10 days postnatal, a markedly reduced number of myelinated axons was observed in mutant mice, in stark contrast to wild-type mice, whose axons were predominantly myelinated by this stage [89]. Although the mutation successfully generated a phenotype resembling a severe and early-onset form of CMT1B, the resultant breeding difficulties render it impractical for widespread use in research. Furthermore, severe symptoms in animal models, even if representative of the disease, could require some ethical considerations. Altogether, this underscores the challenges inherent in developing an accurate disease model for this condition [77].

#### 3.2.6. *Mpz* Q215X Mouse

In 2019, Fratta et al. [63] published the development of a knock-in mouse line using homologous recombination in embryonic stem cells, introducing a nonsense mutation in the mouse *Mpz* gene. The Q215X mutation results in the elimination of a portion of the cytoplasmic domain of MPZ and induces a toxic gain-of-function effect. This mouse line was developed to investigate the mechanisms underlying severely presenting congenital hypomyelination. It was discovered that this mutant Mpz does not activate the UPR and, instead of being retained in the ER, is partially trafficked to non-myelin plasma membranes, disrupting the Schwann cell radial sorting of axons. When examining the g-ratios of *Mpz*+/+, *Mpz*+/−, and Q215X mice, the values were found to be 0.65, 0.70, and 0.69, respectively. Q215X mice also demonstrated reduced performance compared to wild-type mice on the rotarod test. In the grid-walking test, Q215X mice made significantly more errors than wild-type mice. Although these mice exhibited very mild phenotypes, with no overt signs of neuropathy, mild hypomyelination and an increase in unsorted axons were observed in Q215X mice [63].

#### 3.2.7. B6.Cg-*Mpz*^ttrr^/GrsrJ Mouse

The Jackson Laboratory reported a spontaneous mutation in the *Mpz* gene in mouse strain B6.129P2-*Nos3^tm1Unc^*/J, in which exon 8 contains a deletion of 4 nucleotides, as well as a duplication of 12 nucleotides [90]. These mutations lead to multiple amino acid changes, which results in a severe phenotype. Upon characterization, researchers found that these homozygous mice display tremor, limb grasping and a tottering walk, appear hunched and sprawled, and develop late-onset paralysis, leading to premature death. Although phenotypes and pathology varied, mutant mice were also shown to have thinner myelin, hypomyelination, and degeneration of the myelin sheath as well as the dorsal column of the spinal cord [90]. While these mutations in the *Mpz* gene lead to a severe phenotype and disruption of the myelin sheath in the peripheral nervous system, this model is not ideal for use in research due to breeding complications and sporadic death.

### 3.3. Challenges in Modeling CMT1B

Modeling CMT1B is essential for advancing research and therapeutic development. Despite the valuable models discussed above, which have been instrumental in enhancing our understanding of the disease mechanisms, there remains common challenges in modeling Charcot–Marie–Tooth diseases, including CMT1B. Firstly, replicating the complex genetic mutations associated with CMT1B in experimental models is inherently challenging, as these mutations can exhibit varying impacts and expression patterns between animal models and humans. Additionally, recreating the diverse range of symptoms, including muscle weakness, sensory loss, and foot deformities, in model organisms poses a significant challenge. Another major obstacle is the disparity in nerve length and lifespan between humans and animal models, which complicates therapeutic development. Furthermore, comprehending the progressive nature of the disease and its effects on peripheral nerves over time necessitates long-term studies, which can be resource-intensive. These challenges underscore the need for comprehensive approaches that incorporate diverse model systems and consider the complexities of disease progression. Such approaches are essential for developing effective therapies for CMT1B and other peripheral neuropathies, ultimately bridging the gap between preclinical models and human patients [77].

## 4. Current Therapeutic Approaches

Although the molecular mechanisms underlying various types of CMTs were unveiled around three decades ago, and despite significant efforts in developing therapeutic approaches that have shown efficacy in pre-clinical models and some success in clinical stages, there is still no FDA-approved treatment for any type of CMT. Currently, different subtypes are managed symptomatically with supportive care, including pharmacological, surgical, and rehabilitation therapies. These patients require comprehensive care and consideration throughout their lifespan. Pain, fatigue, and cramps are the primary symptoms managed with analgesics, antidepressants, anticonvulsants, and other conventional medications. Physical therapy and exercise can also be beneficial for their overall well-being. In some cases, surgery may be necessary to correct foot deformities and other severe symptoms following thorough evaluations [61,91].

In the pursuit of developing treatments for CMTs, some studies have focused on potential pathways and pathway-specific treatments [15,92,93], which can be beneficial for several subtypes sharing common pathways. However, there remains a considerable need for disease-specific therapeutics. Recent studies highlight gene therapy as the most promising avenue for various types of CMT [91,94]. By addressing the underlying genetic causes, gene therapy offers targeted and effective treatment, providing hope for more precise and long-lasting solutions for patients.

Two primary approaches to develop disease-specific treatments for CMT1B are gene therapy and drug development. While significant progress has been made in pharmaceutical testing, with promising results observed in both preclinical and clinical stages, efforts in gene therapy for CMT1B have yet to emerge. Below, we summarize all efforts related to CMT1B.

### 4.1. Pharmaceuticals

A phase II clinal trial (NCT02967679) sponsored by MedDay Pharmaceuticals SA investigated the efficacy of MD1003 (capsules of biotin 100 mg, three times a day) in demyelinating polyneuropathies such as CMT1A and CMT1B. Outcome measures showed absolute changes from baseline at the end of the study time frame of 48 weeks; however, serious adverse events such as clear cell renal cell carcinoma and autoimmune encephalopathy were reported, requiring further considerations [95]. Furthermore, some active agents including IFB-088, PLX5622, sildenafil, Compound 31, and curcumin, which have different mechanisms, have been posed to alleviate CMT1B symptoms [15,92,93,96,97].

IFB-088, also known as icerguastat and sephin1, has been introduced to prolong the stress response and potentially modulate the UPR by selectively binding to and inhibiting the stress-induced PPP1R15A, while not affecting the related and constitutive PPP1R15B. This action extends the benefits of an adaptive phospho-signaling pathway, thereby protecting cells from potentially fatal protein misfolding stress [98]. This compound exhibited great promise in neurodegenerative diseases such as multiple sclerosis [99]. For CMT1B, sephin1 was shown to improve protein folding efficiency and decrease the expression of ER-stress genes in *Mpz* S63del mutant DRG cultures and mice. Myelin thickness and rotarod activity were also improved in sephin1-treated mice [98]. In another recent study, Bai et al. [15] investigated the efficacy of IFB-088 oral administration in mouse models and demonstrated improvements in various disease symptoms. They evaluated the motor performances of *Mpz* R98C mice using grip strength and rotarod assays and reported an improvement in heterozygous animals following treatment. Additionally, they observed improvements in both MNCV and sensory nerve conduction velocity (SNCV) in heterozygous *Mpz* R98C models; however, they did not detect any significant differences in CMAP compared to wild-type mice [15].

Another molecule, PLX5622, demonstrated the potential to alleviate peripheral neuropathy when orally administered early within a critical time window in *Mpz* null mice. Treatment groups exhibited improvements in motor performance; however, the CMAP and MNCV results did not show significant improvement, necessitating further investigation. The active agent depletes nerve macrophages, which are key players in disease progression and severity [93].

In an alternative approach, a marketed drug was repurposed to investigate the potential effect of increasing cyclic guanosine monophosphate (cGMP) level in *Mpz* S63del mice. Proteasome activities are crucial for degrading misfolded and damaged proteins, thereby preventing subsequent cellular stress in neurodegenerative diseases. Proteasome activity is decreased in CMT1B, leading to disease symptoms. Increasing cGMP levels aids in the activation of proteasomal activities. By this rationale, sildenafil, a phosphodiesterase 5 inhibitor, was injected intraperitoneally to decrease the number of amyelinated axons in sciatic nerves and improve myelin thickness and conduction velocity by raising cGMP levels via the nitric oxide pathway [96].

Another treatment approach was proposed based on an abnormal expression of Na_V_1.8, a sodium channel isoform specific for sensory neurons, on motor axons. An oral Na_V_1.8 channel blocker, Compound 31, was orally administered in *Mpz* null mice, and it was indicated that motor function improved in severe forms of CMT [92]. Furthermore, Shy and coworkers explored the use of curcumin derivatives to improve neuropathy symptoms. In the cell culture, curcumin was found to reduce apoptosis by releasing ER-retained MPZ mutants into the cytoplasm [64]. Building on these findings, oral administration of phosphatidylcholine curcumin or curcumin dissolved in sesame oil in an *Mpz* R98C mouse model demonstrated partial improvements in peripheral neuropathy in heterozygous mice. The treated mice demonstrated enhanced phenotypes, including increased CMAP amplitudes, larger axon diameters, and better performance on the rotarod. However, there was no improvement in nerve conduction velocity, myelin thickness, g-ratios, or myelin periodicity. The beneficial effect of curcumin involves alleviating endoplasmic reticulum stress, reducing the activation of the unfolded protein response, and promoting Schwann cell differentiation. Despite these positive outcomes, curcumin did not enhance MNCV, myelin thickness, g-ratios, or myelin periodicity [97].

### 4.2. Gene Therapies

Gene therapy offers a promising approach for treating CMT1B, providing the potential for a one-time treatment with lifelong therapeutic benefits that directly target the genetic cause of the disease. Despite this potential, no gene therapy efforts have been made for CMT1B until now. As explained in previous sections, most mutations in the *MPZ* gene cause disease symptoms through toxic gain-of-function mechanisms [3,7,8,45,46]. Therefore, depleting the mutant protein is crucial to alleviate the cellular burden it causes. On the other hand, MPZ is a major protein of the myelin sheath, so replacing it with a healthy copy is essential to maintaining a functional myelin sheath. Our team has pioneered a novel gene therapy strategy known as KnockDown And Replacement (KDAR) for CMT1B [84]. This method involves using RNA interference (RNAi) to knock down the mutant *MPZ* gene and replacing it with an RNAi-resistant *MPZ* (r*MPZ*) gene. This strategy has been successfully employed in models of other diseases, including CMT2A [21], autosomal dominant retinitis pigmentosa [100], oculopharyngeal muscular dystrophy [101,102], and spinocerebellar ataxia 7 [103]. Ex vivo testing of this approach in *Mpz* R98C DRG explants showed a significant reduction in mutant *Mpz* and ER stress biomarker expression levels, alongside a notable increase in r*MPZ* expression. This was accompanied by a rise in MBP-positive internodes. Studies on our AAV-based KDAR approach in vivo are ongoing [84].

In another study, researchers investigated the effect of mesencephalic astrocyte-derived neurotrophic factor (MANF) on ER stress in RT4-D6P2T schwannoma cells with the S63del *Mpz* mutation. They used lentivectors to deliver *MANF* into these cells in vitro. The results showed that while the S63del *Mpz* mutation led to decreased cell proliferation and increased apoptosis, *MANF* treatment improved proliferation and reduced apoptosis, indicating its protective role [68]. This research suggests that MANF could be a promising therapeutic approach for managing ER stress in CMT1B; however, additional in vivo proof-of-concept studies are necessary to further validate these findings. CRISPR genome editing would be another potential therapeutic approach for CMT1B, but because of various mutations in the MPZ gene and the autosomal dominant nature of the disease, this approach might be much more challenging.

## 5. Challenges and Future Perspectives

Despite significant progress in understanding the disease mechanisms and successful preclinical testing of some pharmaceuticals, there are still no FDA-approved treatments for CMT1B. Until now, several challenges have hindered the translation of CMT1B preclinical findings to clinical applications. One major challenge is the slow progression and phenotypic variability of the disease, which limits the evaluation of treatment responses over short periods. To address this, the development of novel biomarkers is essential to facilitate the optimization of potential CMT1B clinical trials [5,104]. Recent studies suggested neurofilament light chain (NFL) as a CMT disease biomarker. In a recent report in 2021, plasma NFL concentration was increased in a US cohort of CMT patients including CMT1B, compared with healthy controls [105]. In another study, transmembrane protease serine 5 (TMPRSS5) was suggested as a Schwann cell-specific biomarker elevated in CMT1A plasma [106]. This protein was also investigated in CMT1B samples; however, data showed a modest increase lacking statistical significance. This could be due to the wide range of CMT1B mutations and phenotype severity [106].

In another study, elevation in several muscle-associated miRNAs, also known as myomiRNAs, including miR-1, miR-133a, miR-133b, and miR-206, as well as Schwann cell-specific miRNAs such as miR-133a, miR-206, and miR-223, was detected in CMT1A patients compared to control samples. Among the myomiRNAs, miR-133a was the most highly correlated with NFL levels. In contrast, Schwann cell miRNAs showed a correlation with TMPRSS5 [107]. Investigating the elevation of these miRNAs in CMT1B and other CMT patients is crucial for understanding the underlying molecular mechanisms and could potentially lead to the development of novel diagnostic and therapeutic strategies. In addition to molecular biomarkers, quantifying fat fractions of the calf muscle via magnetic resonance imaging (MRI) has emerged as another potential CMT disease biomarker [108]. The pattern of fatty infiltration has been shown to correlate with CMT subtype; however, all CMT1 patients in this study were from the CMT1A subtype [108]. Further investigations are necessary to identify more CMT1B-specific biomarkers, which facilitate monitoring of disease progression and treatment response for future therapeutic evaluations. Additionally, by employing a comprehensive set of multiple biomarkers, the success rate of clinical trials may be significantly improved.

Another challenge in designing clinical trials for CMT1B lies in the variable disease severity across the three major onset stages—infantile, childhood, and adult. Conducting natural history studies can significantly enhance the design of clinical trials tailored to each group [109,110,111]. Additionally, the complex disease mechanism of CMT1B, which is driven by over 200 mutations in the *MPZ* gene, presents another significant challenge. This review summarizes the multiple mechanisms involved in CMT1B pathogenesis.

Several groups are exploring various therapeutic solutions for CMT1B disease, including mechanism-based pharmaceutical therapy [15,92,93,96,97] and gene therapy [68,84]. Each approach aims to address different aspects of the disease’s pathology, offering multiple potential strategies for effective treatment. Since pharmaceutical agents target specific pathways, several drugs need to be developed to address the diverse pathomechanisms within the CMT1B patient population. Accordingly, some potential small molecules were introduced and discussed in the pharmaceuticals section of this paper [15,92,93,96,97]. Researchers are still trying to optimize and target these drug candidates.

Additionally, there are some recent publications on the application of stem cells in CMT1A disease models and a CMT patient case, suggesting improved phenotypes [112,113]. It was reported that a 19-year-old male patient was successfully treated with the Regentime procedure, an autologous bone marrow mononuclear progenitor stem cell transplantation via intrathecal and intravenous routes. The sensation and motor power of the patient gradually improved, resulting in unassisted walking at 10 months post-treatment. In addition, his overall neuropathy limitations scale (ONLS) dropped from 4/12 to 0/12 [113]. In another study on a CMT1A mouse model, murine adipose-derived mesenchymal stromal cells (AD-MSCs) were transplanted into triceps surae (TS) muscles. Three months after transplantation, the force generated by TS muscle contraction was observed to be twice as high in the treated group compared to untreated mice [112]. However, it is worth noting that mechanism- and phenotype-specific therapies can alleviate the symptoms of the disease, but often do not provide a long-term therapeutic response.

Conversely, genome editing and gene therapy approaches offer the possibility of long-term, and ideally life-long, solutions. CRISPR-Cas9-mediated genome editing has been applied to CMTs, particularly CMT1A [114]. However, in the context of CMT1B, although genome editing may be considered an attractive option, it poses significant challenges due to the extensive number of mutations associated with the *MPZ* gene. This necessitates the design of multiple sets of guide RNAs (gRNAs) to accurately target each specific mutation. Consequently, more extensive preclinical and clinical trials are required to develop mutation-specific therapies capable of treating all patients. Moreover, since most of the mutations in the *MPZ* gene are missense or small indel mutations, precisely targeting these specific nucleotide changes adds an additional layer of complexity to the development of effective treatments. In contrast, mutation-independent gene therapy strategies, such as KDAR therapy [84], offer the advantage of potentially serving as a universal treatment for all patients. This strategy has been successfully employed in models of other diseases, including CMT2A [21], autosomal dominant retinitis pigmentosa [100], oculopharyngeal muscular dystrophy [101,102], and spinocerebellar ataxia 7 [103].

For many years, a significant obstacle in gene therapy development for demyelinating diseases has been the delivery of therapeutic cassettes to Schwann cells in peripheral nerves. However, recent studies have demonstrated considerable progress in this area, successfully targeting Schwann cells in rodent models using AAV9 and AAVrh10 vectors [22,23,24,25,26,115]. It was reported that 30% of cells in the lumbar roots were enhanced green fluorescent protein (EGFP)-positive following intrathecal injection of AAV9 and AAVrh10 vectors in mice. Additionally, in the sciatic nerves, 53% of cells were EGFP-positive with AAV9, compared to 39% with AAVrh10 [115]. This level of transduction was sufficient to achieve therapeutic efficacy in mice. To further support gene therapy clinical trials for CMT1B and other demyelinating neuropathies, AAV capsid engineering could offer an effective solution by developing Schwann cell tropic AAVs [116]. Finally, lessons learned from clinical trials for other types of CMT could serve as valuable guides for the development of future studies and trials in CMT1B.

## Figures and Tables

**Table 1:** Summary of in vitro CMT1B disease models.

Model	Mutations	Advantages	Disadvantages	References
HEK293, HeLa, RT4, etc.	R98C, S63del, T65D, D118N	Commercially available, easy to culture, well characterized	Not necessarily representative of the physiology of tissues of interest, transient protein expression	[14,45,62,64]
RT4-D6P2T	S63del	Protein expression over several generations	Not necessarily representative of the physiology of tissues of interest	[68]
iPSCs	R98C, T140S	Ability to differentiate into nearly any cell type, patient-specific genotype	Technically challenging to culture, potential interference of mutations with differentiation	[66,67]
DRG explant/Schwann cell co-cultures	R98C, S63del, D61N	Recapitulation of neuron environment, useful in myelination studies	Technically challenging to isolate and culture	[15,16,17,45,56,57]

Abbreviations: DRG: dorsal root ganglia; iPSCs: induced pluripotent stem cells.

**Table 2:** Summary of CMT1B mouse models.

Model Type	Mutation	Affected Domain	Inheritance Pattern	Pathomechanisms	Phenotype				References
					MNCV (m/s)	CMAP (mV)	G-Ratio	Other	
Knock-out	Null (P0−/−. P0+/−)	Extracellular, transmembrane, cytoplasmic	Dominant	Loss of adhesion properties	-	-	-	Tremor, reduced muscle strength, defective myelin compaction	[86,87,88]
Knock-in	R98C	Extracellular	Dominant	ER retention, UPR activation	~15	~10	0.77	Tremor, unsteady gait, thinner myelin, defective myelin compaction	[12,13,15,16]
Transgenic	S63del	Extracellular	Dominant	ER retention, UPR activation, ER stress	~24	~8.2	0.69	Unsteady gait, thinner myelin, muscle atrophy, onion bulb formation	[7,16,17,18,19,20,51,53,56]
Transgenic	D61N	Extracellular	Dominant	Gain of glycosylation	~12.05	~0.2	0.72	Tremor, reduced muscle strength, defective myelin compaction, reduction in myelinated axon diameter	[45]
Transgenic	I106L	Extracellular	Dominant	-	~2	~12.6	-	Tremor, dispersed action potentials, onion bulb formation, tomacula in nerve fibers, severe muscle weakness, dragging limbs	[89]
Knock-in	Q215X	Cytoplasmic	Dominant	Mistrafficking to non-plasma membranes	-	-	0.69	Defective axonal radial sorting, mild hypomyelination, no external phenotype	[63]
Congenic	ttrr	Extracellular	Recessive	-	-	-	-	Tremor, severe muscle weakness, tottering walk, thinner myelin, hypomyelination, hyperproliferation of Schwann cells, degeneration of dorsal column	[90]

Abbreviations: CMAP: compound muscle action potential; ER: endoplasmic reticulum; MCNV: motor nerve conduction velocity; UPR: unfolded protein response.
